# The Active Astrocyte: Calcium Dynamics, Circuit Modulation, and Targets for Intervention

**DOI:** 10.1007/s11064-025-04553-1

**Published:** 2025-09-27

**Authors:** Dmitri A. Rusakov, Thomas P. Jensen, Olga Tyurikova

**Affiliations:** UCL Queen Square Institute of Neurology, University College London, London, United Kingdom

**Keywords:** Tripartite synapse, Astrocyte calcium microdomains, Gliotransmission

## Abstract

Astrocytes, once considered passive support cells, have emerged as active participants in synaptic communication through Ca^2+^-dependent molecular signalling often referred to as gliotransmission. This review highlights the pioneering contributions of Giorgio Carmignoto, whose work has helped to redefine astrocytes as integral components of the tripartite synapse. Central to this paradigm shift is the role of astrocytic Ca²⁺ signalling in modulating synaptic activity, plasticity, and network behaviour. Carmignoto’s research demonstrated that intracellular Ca^2+^ fluctuations in astrocytes trigger the release of signalling molecules, influencing both excitatory and inhibitory neuronal circuits. These discoveries extended to network-level phenomena, implicating astrocytic Ca^2+^ waves in pathological states like epilepsy. Technologically, Carmignoto advanced astroglial research by employing genetically encoded calcium indicators, optogenetic tools, and cutting-edge imaging methods, including multi-photon microscopy, to observe astrocyte activity in vivo. His work also contributed to automated data analysis pipelines that uncover fine-scale astrocytic microdomain dynamics. In the context of pathology, Carmignoto’s studies related astrocytic dysfunction to epilepsy and dopaminergic dysregulation, suggesting new therapeutic avenues through astrocyte-specific interventions. Despite these advances, challenges remain in defining gliotransmitter mechanisms, understanding astrocyte heterogeneity, and developing tools for precise functional manipulation.

## Introduction

Historically, astrocytes were regarded as passive elements in the central nervous system, responsible primarily for maintaining ionic balance, providing metabolic support, and acting as structural scaffolds. This traditional view has shifted dramatically with the recognition of the tripartite synapse, wherein astrocytes actively participate in various aspects of neuronal signalling. This model, which incorporates astrocytes alongside pre- and postsynaptic neurons, has profoundly altered our understanding of synaptic communication and plasticity [1, 2]. Among the key figures in this conceptual transformation is Dr. Giorgio Carmignoto, whose research has focused on astrocytic Ca^2+^ signalling as a central mechanism underlying gliotransmission, the release of neurotransmitter-like substances from astrocytes. His work has demonstrated how astrocytes dynamically regulate synaptic activity, plasticity, and network-level behaviours. A recurrent theme in his studies is the bidirectional communication between astrocytes and neurons, a relationship once considered ancillary but now recognised as essential for synaptic integration and circuit homeostasis. Importantly, Carmignoto’s investigations have linked astrocytic Ca^2+^ dynamics to major neuropathological conditions, particularly epilepsy.

This brief review synthesises the major research areas influenced by Carmignoto’s contributions, including astrocytic Ca^2+^-dependent gliotransmission, interneuron-astrocyte interactions, vascular regulation, astrocytes in epilepsy, and technological innovations that have enabled their study. We conclude with a perspective on current challenges and future directions in astroglial research.

## Astrocytic Ca^2+^ Oscillations and Gliotransmission

Astrocytes exhibit a non-electrical form of excitability based on intracellular Ca^2+^ fluctuations rather than regenerative action potentials. Carmignoto was among the first to demonstrate that elevations in astrocytic Ca^2+^ are tightly coupled to the release of signalling molecules such as the excitatory neurotransmitter glutamate [3–5] or prostaglandins [6]. He and his colleagues showed that astrocytic Ca^2+^ oscillations constitute a core mechanism for functional signalling between astrocytes and neurons [7, 8].

His group further demonstrated the involvement of purinergic receptor signalling in generating astrocytic glutamate release [9], which could in turn synchronise neuronal firing via the activation of extrasynaptic NMDA receptors [10]. This and subsequent works introduced the concept of a multifunctional neuron-astrocyte signalling unit [11]. Importantly, their studies reveal a diversity of astrocytic glutamate-release mechanisms, by showing that Ca^2+^-dependent release appears distinct from glutamate release events that generate slow inward currents (SICs) in neurons [12]. The origin, conditions, and prevalence of SICs, however, remain debatable [13, 14]. Nonetheless, these and related studies established the principle that astrocytes are not passive, but active, modulators of neuronal communication.

## Interneuron–Astrocyte Communication

Beyond excitatory transmission, Carmignoto demonstrated that astrocytes also respond dynamically to GABAergic signalling. His group found that the inhibitory neurotransmitter GABA is a potent trigger of astrocytic Ca^2+^ oscillations [15, 16]. More specifically, they showed that somatostatin-positive interneurons evoke distinct Ca^2+^ responses in cortical astrocytes, exhibiting use-dependent plasticity [17]. This signalling may also involve astrocytic GABA transporters [18], has broad implications for the interaction of GABAergic networks with astrocytic ensembles [19], As the balance between phasic (fast synaptic) and tonic (slow, both synaptic and extrasynaptic) GABAergic signals appears critically involved in regulating epileptogenesis, a mouse model of Dravet syndrome helped the group to unveil an important role of astrocytes in the underlying machinery [20].

## Impact on Astroglial and Neuronal Plasticity

A landmark study by Carmignoto group demonstrated activity-dependent long-term changes in astrocytic activity, suggesting that non-neuronal cells may encode a form of memory trace [21], an idea corroborated by subsequent independent studies [22, 23]. They later expanded these findings to the plasticity-triggering mechanisms of inhibitory network activity, revealing key intracellular pathways [16, 17].

More recently, Carmignoto’s group showed that astrocytic Ca^2+^ signalling mediated by endocannabinoid CB1 receptors and dopamine D2 receptors, which are co-localised at astrocytic processes, induces long-lasting potentiation of excitatory synapses onto dopamine neurons, which also involves presynaptic mGluR activation [24]. In a female mouse model of Alzheimer’s disease, his team demonstrated that restoring astrocytic activity by rescuing the Ca^2+^ sensor STIM1 could normalise impaired long-term synaptic plasticity [25].

While the sheer diversity of molecular cascades triggered by Ca^2+^ rises in astrocytes raises questions about how they achieve specificity in particular contexts, these findings do challenge neuron-centric models of plasticity by positioning astrocytes as activity-dependent modulators of synaptic strength. In many such cases, simultaneous monitoring of astrocyte and neuronal activity reveal that astrocytic Ca^2+^ oscillations can follow and influence neuronal firing patterns, functioning as a possible feedback mechanism for circuit modulation [26].

## Astrocytes and Vascular Regulation

Another important contribution of Carmignoto’s work concerns neurovascular coupling. Early studies of the group pointed to reciprocal signalling between astrocytes and neurons mediated by prostaglandins [5, 6], thereby suggesting a potential link of astrocytic activity to cerebral blood flow regulation, which they have subsequently demonstrated [27].

Carmignoto and colleagues later showed that ictal discharges activate astrocytic endfeet, eliciting vasomotor responses in cerebral arterioles [28]. These findings, later expanded by others [29], established astrocytes as important regulators of brain vasculature [11, 26, 30]. This line of work widened the significance of astrocytes beyond synaptic regulation, to include important influences in maintaining metabolic environment and energy homeostasis of the brain.

## Astrocytes in Epilepsy

Carmignoto’s group obtained some of the early evidence that astrocytic Ca^2+^ signalling is involved in epileptiform activity. They showed that synchronized Ca^2+^ oscillations in astrocytes often precede and sustain seizure events in experimental models [31]. Importantly, these Ca^2+^signals were not passive responses but appear to advance seizure onset by regulating local neuronal excitability [31–33].

While their initial studies showed that astrocytic glutamate release was not necessary for generation of epileptiform activity [34], later findings from the group demonstrated that high interneuron activity drives GABA-induced astrocytic Ca^2+^ oscillations, boosting network excitation through glutamate release [35, 36]. Subsequent work linked astrocytic glutamate release to enhanced neuronal synchrony via activation of extrasynaptic NMDA receptors [10], and to neuronal excitotoxicity after status epilepticus [37]. In addition, Garmignoto team reported alterations in GABAergic tonic currents and astrocytic signalling during epileptogenesis in Dravet syndrome models [20]. Together, these findings underscore the dual role of astrocytes in epilepsy, as drivers of pathological hyperexcitability and as potential therapeutic targets [31, 33, 38], the concept corroborated by subsequent studies [39–42].

## Tools and Methodological Advances

The impact of Carmignoto studies extends beyond scientific discovery to the development or refinement of experimental methodologies that enabled direct study of astrocytic dynamics in vivo and ex vivo. His group pioneered several task-specific synchronous bioimaging approaches, including intracellular pH and chloride monitoring based on LSS fluorescent proteins [43]. They also developed brain slice models to study focally induced epileptiform activity [44, 45], which provided well-controlled systems for investigating seizure initiation and propagation.

Further innovations include the application of task-targeted genetically encoded calcium indicators, optogenetic actuators, and chemogenetic tools, which permitted cell-specific manipulation of astrocytic activity. With these approaches, Carmignoto group could relate the dynamics of astrocytic Ca^2+^ transients to neuronal and behavioural outcomes [17, 46, 47]. They continued to advance optical methods such as two-photon microscopy and chronic cranial windows allowing long-term, high-resolution imaging of astrocytic processes and vascular interactions in intact brain tissue. These methodological contributions have been essential in revealing the fine-scale and dynamic nature of astrocytic signalling.

## Current Challenges

Despite major advances, several conceptual and methodological challenges remain. First, the identity and regulation of gliotransmitters is still debated. While astrocytes can release glutamate, ATP, and dD-serine in response to Ca^2+^ signals, the conditions under which the release of a particular molecule predominates remain unclear [48–50]. Similarly, the precise release mechanisms, vesicular versus non-vesicular (e.g., hemichannels, Bestrophins), are incompletely defined and may vary across regions and developmental stages. Electron micrographs reveal that the number of synaptic glutamatergic vesicles in neurons vastly outnumbers, by several orders of magnitude, those found in astrocytes. This disparity raises questions about the comparative physiological impact of vesicular glutamate release from astrocytes versus neurons. Moreover, astrocyte membranes are densely populated with high-affinity glutamate transporters, particularly GLT-1 [51], which appear to cover the entire cell surface [52]. This dense transporter pattern suggests that the effects of astrocyte-released glutamate are likely as spatially restricted as those of excitatory synapses. One theoretical possibility that could account for more extensive astrocyte-driven glutamate signalling is a localised failure of their glutamate transporters, leading to either a massive unbinding or an incomplete binding of glutamate molecules.

Secondly, the temporal precision problem persists as neuronal firing occurs on the millisecond scale, whereas astrocytic Ca^2+^ events are slower, although the latter could be, at least in part, a reflection of the high affinity of available Ca^2+^ sensors [53]. One proposed solution is that astrocytic modulation operates via local microdomains, enabling rapid and spatially restricted gliotransmission at perisynaptic sites. Indeed, the assumption that the Ca^2+^ release machinery that engages astrocytic internal Ca^2+^ stores resembles the classical “sparks-and-puffs” mechanisms established in other cell types [54, 55] is, at least theoretically, consistent with experimentally observed astrocytic Ca^2+^ dynamics on the sub-micron scale [56]. Elucidating the biophysical properties of these microdomains, the potential diversity in their signalling dynamics, and the consequences of their specific signals for local network activity remains a pressing objective.

Thirdly, astrocyte heterogeneity presents a universal challenge to unified models. Recent studies revealed that astrocytes differ substantially between cortical, hippocampal, and striatal regions in terms of gene expression, electrophysiology, and Ca^2+^ dynamics [57–59]. Whether a single conceptual framework can capture this diversity, or whether astrocyte function must be understood in a region-specific manner, is unresolved. Single-cell RNA sequencing and spatial transcriptomics, when combined with advanced imaging approaches, might be able to somewhat clarify these questions, although to what extent such “snapshots” could capture the temporal dynamics of cell diversity remains unclear.

Finally, the therapeutic potential of astrocyte modulation is becoming increasingly tangible. Astrocyte-targeted strategies, ranging from pharmacological modulation of Ca^2+^ signalling, K^+^ homeostasis and glutamate clearance, to gene therapy vectors directed at GFAP-positive cells, are now under investigation in models of epilepsy, gliosis, and neurodegeneration. However, to what degree the experimental manipulations of astrocyte function developed and tested in animal models could represent a feasible path to human therapy remains to be seen. In any case, Carmignoto’s studies have contributed significantly to the foundation for such translational advances, positioning astrocytes as promising targets for future treatments.

## Concluding Remarks

Dr. Giorgio Carmignoto’s body of work has helped to reshape the field of glial biology. His studies provided critical contributions to establishing astrocytes as active, bidirectional regulators of synaptic function, plasticity, and network behaviour, while implicating their dysfunction in pathological states such as epilepsy. His methodological innovations have furthered and encouraged inter-disciplinary studies of astrocytic activity, while his mechanistic insights continue to inspire both fundamental research and translational approaches. As the field advances, addressing unresolved questions of gliotransmission-trigger identity, spatiotemporal precision of Ca^2+^ signalling, and potentially fluid astrocytic heterogeneity will be critical.

## Data Availability

No datasets were generated or analysed during the current study.
